# Newly Reported Chronic Hepatitis C Among Adults — Alaska, 2016–2023

**DOI:** 10.15585/mmwr.mm7410a1

**Published:** 2025-03-27

**Authors:** Heather M. Scobie, Jamie Allison, Nicholas Masters, Morrow Toomey, Ian Blake, Janet M. Johnston, Eyasu Teshale, Robert Lawrence, Elizabeth Ohlsen, Dana Bruden, Marc Fischer, Joe McLaughlin

**Affiliations:** ^1^Arctic Investigations Program, Division of Infectious Disease Readiness and Innovation, National Center for Emerging and Zoonotic Infectious Diseases, CDC; ^2^Section of Epidemiology, Alaska Department of Health, Anchorage, Alaska; ^3^Liver Disease and Hepatitis Program, Alaska Native Tribal Health Consortium, Anchorage, Alaska; ^4^Division of Viral Hepatitis, National Center for HIV, Viral Hepatitis, STD, and Tuberculosis Prevention, CDC; ^5^Alaska Department of Corrections, Anchorage, Alaska.

**Summary**

**What is already known about this topic?**

Hepatitis C is a substantial cause of morbidity and mortality and is targeted for global elimination by 2030. All adults should receive hepatitis C virus testing, and persons with evidence of current infection should receive antiviral treatment.

**What is added by this report?**

In Alaska, the average annual rate of newly reported chronic hepatitis C (cases per 100,000 adults) during 2016–2023 was 121; the rate decreased a relative 30% from 142 (2016–2019) to 99 (2020–2023). Rates decreased for most groups but were higher overall among males, persons aged 18–39 years, residents of rural areas, and American Indian or Alaska Native persons.

**What are the implications for public health practice?**

Continued hepatitis C surveillance can help identify groups needing tailored testing and treatment interventions toward hepatitis C elimination.

## Abstract

Hepatitis C virus is a leading cause of chronic liver disease, hepatocellular carcinoma, and liver-related death and is targeted for global elimination as a public health threat by 2030. Universal screening is recommended for all adults aged ≥18 years and pregnant women during each pregnancy; periodic risk-based screening also is recommended. Persons with current infection should be linked to antiviral treatment, which usually results in a virologic cure within 8–12 weeks. To assess progress toward elimination, epidemiologic trends in newly reported chronic hepatitis C cases were assessed among adult Alaska residents during 2016–2023. Overall, 5,352 confirmed chronic hepatitis C cases were newly reported among adults aged ≥18 years. The average annual rate (cases per 100,000 population) was 121 and decreased a relative 30% from 142 during 2016–2019 to 99 during 2020–2023. Statistically significant decreases occurred for most groups. Groups with higher average rates included males, adults aged 18–39 years, residents of rural areas, and American Indian or Alaska Native persons. Hepatitis C surveillance can help monitor trends in health outcomes and identify groups needing tailored testing and treatment interventions toward hepatitis C elimination.

## Introduction

Hepatitis C virus (HCV) is a leading cause of chronic liver disease, hepatocellular carcinoma, and liver-related death and is targeted for elimination as a public health threat by 2030 (*1*).* In the United States, an estimated 2.2 million adults had current HCV infection during 2017–2020 (*1*). Hepatitis C incidence and mortality have disproportionately affected American Indian or Alaska Native persons (*2*). Before 2009, hepatitis C risk was highest among persons born during 1945–1965, but recent increases have occurred among young adults in association with increased injection drug use (*3*,*4*). In 2014, direct-acting antiviral treatments for HCV infection became available that result in >95% virologic cure among persons who complete treatment (*4*,*5*). In 2020, CDC recommended universal hepatitis C screening of all adults aged ≥18 years (at least once during their lifetime) and pregnant women during each pregnancy, with subsequent linkage to care for those with current infection; periodic risk-based screening also is recommended (*4*). To assess progress toward elimination, trends in newly reported chronic hepatitis C cases were assessed among adult Alaska residents during 2016–2023.

## Methods

Trends in chronic hepatitis C cases among Alaska residents aged ≥18 years during 2016–2023 were analyzed using data from the National Electronic Disease Surveillance System Base System^†^ reported to the Alaska Department of Health as of May 21, 2024. A confirmed case of chronic hepatitis C was defined as 1) a positive test result for HCV RNA, 2) no documentation of converting from negative to positive anti-HCV antibody or HCV RNA test results within the previous 12 months, and 3) not meeting the criteria for or having no report of clinical signs or symptoms of acute hepatitis (i.e., jaundice, total bilirubin ≥3 mg/dL, or serum alanine aminotransferase >200 IU/L).^§^

The numbers and percentage of newly reported chronic hepatitis C cases were analyzed by sex, age group, residential area (urban, rural, and remote),^¶^ public health region, race, ethnicity, and year that the first positive test result for HCV RNA or antibody was reported. Annual rates were calculated using population estimates from the Alaska Department of Labor and Workforce Development.** Direct age-standardization was performed using U.S. Census Bureau 2020 data^††^ as a reference population. CIs were estimated using a normal approximation for age-specific rates and a gamma approximation of the Poisson distribution for age-standardized rates. The same method was used to calculate the relative percentage change in age-standardized rates from 2016–2019 to 2020–2023. Data were analyzed using SAS (version 9.4; SAS Institute). This activity was reviewed by CDC, deemed not research, and was conducted consistent with federal law and CDC policy.^§§^

## Results

During 2016–2023, a total of 5,352 confirmed cases of chronic hepatitis C among adults aged ≥18 years were newly reported in Alaska (Table 1). A majority of the cases were among males (66%) and adults aged <40 years (61%). Overall, 25% of cases were reported among women of reproductive age (18–44 years). Median age of newly reported cases was 38 (IQR = 29–54) years in 2016 and 35 (IQR = 30–45) years in 2023.

**TABLE 1:** Number, percentage, and average age-standardized rate of newly reported chronic hepatitis C cases per 100,000* adults aged ≥18 years, by sociodemographic group — Alaska, 2016**–**2023^†^

Characteristic	No. (%)	Rate (95% CI)
**Overall**	**5,352 (100)**	**121 (118–124)**
**Sex**		
Female	1,837 (34)	86 (82–90)
Male	3,515 (66)	153 (148–159)
**Age group, yrs**		
18–29	1,524 (28)	160 (152–168)
30–39	1,746 (33)	196 (187–206)
40–49	739 (14)	106 (98–113)
50–59	666 (12)	90 (83–96)
≥60	677 (13)	60 (55–64)
**Area^§^**		
Rural	1,480 (28)	162 (154–170)
Urban	2,884 (54)	110 (105–114)
Remote	917 (17)	104 (97–111)
Unknown	71 (1)	NC
**Region**		
Southeast	686 (13)	156 (144–168)
Gulf Coast	705 (13)	154 (142–165)
Matanuska-Susitna	913 (17)	153 (144–163)
Anchorage	2,158 (40)	118 (113–124)
Southwest	283 (5)	114 (100–128)
Interior	439 (8)	64 (58–70)
Northern	97 (2)	57 (45–69)
Unknown	71 (1)	NC
**Race^¶^**		
AI/AN	1,856 (35)	223 (213–233)
Asian	69 (1)	19 (14–23)
Black or African American	205 (4)	101 (87–116)
NH/PI	51 (1)	22 (4–39)
White	2,521 (47)	78 (75–81)
Other	265 (5)	NC
Unknown	498 (9)	NC
Ethnicity**		
Hispanic or Latino	202 (4)	67 (57–76)
Not Hispanic or Latino	4,106 (77)	100 (97–104)
Unknown	1,044 (20)	NC

**Abbreviations:** AI/AN = American Indian or Alaska Native; NC= not calculated; NH/PI = Native Hawaiian or Pacific Islander.

* Annual rates were calculated using population estimates from the Alaska Department of Labor and Workforce Development. Direct age-standardization was performed using U.S. Census Bureau 2020 data as a reference population. Age-specific rates were not age-standardized.

^†^ All data are current as of May 21, 2024, and are subject to change, pending further updates.

^§^ Urban was defined as residence in the Municipality of Anchorage, the Fairbanks North Star Borough, or the City and Borough of Juneau. Rural was defined as living in the Matanuska-Susitna or Kenai Peninsula Boroughs, which are on the road system. All other areas were considered remote.

^¶^ For race, categories are not mutually exclusive: 112 (2%) persons selected two or more racial groups and are counted more than once in the analysis by racial group.

** Race and ethnicity were reported separately and are not mutually exclusive (i.e., persons reporting Hispanic or Latino ethnicity could have identified as any race).

During 2016–2023, the average annual age-standardized rate of newly reported chronic hepatitis C cases in Alaska was 121 per 100,000 adults (Table 1). By age group, rates were highest among adults aged 30–39 years (196) and 18–29 years (160) and lowest among those aged ≥60 years (60). By location, rates were highest among adults living in rural areas (162) and among residents of Alaska’s Southeast (156), Gulf Coast (154), and Matanuska-Susitna (153) regions. Average rates by race were highest among American Indian or Alaska Native adults (223).

The rate of newly reported chronic hepatitis C per 100,000 adults decreased a relative 30% (95% CI = 26%–35%) from 142 during 2016–2019 to 99 during 2020–2023 (Table 2) (Figure). From 2016–2019 to 2020–2023, a statistically significant decrease in chronic hepatitis C rates occurred among all age groups except among persons aged 40–49 years. A significant decrease occurred in all regions except the Southwest and Northern regions. A decrease in the chronic hepatitis C rate occurred among American Indian or Alaska Native (−20%), Asian (−45%), White (−26%), and non-Hispanic or Latino persons (−25%). No group experienced a significant increase in chronic hepatitis C.

**TABLE 2:** Average age-standardized rate* of newly reported hepatitis C cases per 100,000 adults aged ≥18 years and percentage change,^†^ by sociodemographic group — Alaska, 2016–2019 and 2020–2023^§^

Characteristic	2016–2019		2020–2023		Relative % change (95% CI)
	No (%)	Rate (95% CI)	No (%)	Rate (95% CI)	
**Overall**	**3,198 (100)**	**142 (137 to 147)**	**2,154 (100)**	**99 (95 to 103)**	**–30 (−35 to −26)**
**Sex**					
Female	1,129 (35)	104 (98 to 111)	708 (33)	68 (63 to 73)	−35 (−43 to −27)
Male	2,069 (65)	178 (170 to 185)	1,446 (67)	129 (122 to 135)	−27 (−33 to −22)
**Age group, yrs**					
18–29	976 (31)	199 (186 to 211)	548 (25)	118 (108 to 128)	−40 (−48 to −33)
30–39	938 (29)	215 (201 to 229)	808 (38)	178 (166 to 191)	−17 (−26 to −8)
40–49	393 (12)	113 (102 to 125)	346 (16)	98 (88 to 108)	−13 (−28 to 1)
50–59	442 (14)	112 (102 to 123)	224 (10)	64 (56 to 72)	−43 (−55 to −31)
≥60	449 (14)	85 (77 to 93)	228 (11)	38 (33 to 43)	−55 (−67 to −44)
**Area^¶^**					
Rural	899 (28)	200 (186 to 213)	581 (27)	125 (115 to 135)	−37 (−46 to −29)
Urban	1,752 (55)	129 (123 to 136)	1,132 (53)	89 (84 to 94)	−31 (−37 to −25)
Remote	517 (19)	115 (104 to 125)	400 (19)	94 (84 to 103)	−18 (−30 to −6)
Unknown	30 (1)	NC	41 (2)	NC	NC
**Region**					
Southeast	386 (12)	168 (151 to 186)	300 (14)	144 (127 to 160)	−15 (−29 to 0)
Gulf Coast	455 (14)	197 (179 to 216)	250 (12)	110 (96 to 124)	−44 (−56 to −32)
Matanuska-Susitna	540 (17)	184 (169 to 200)	373 (17)	123 (110 to 135)	−33 (−44 to −23)
Anchorage	1,313 (41)	140 (133 to 148)	845 (39)	96 (89 to 103)	−32 (−39 to −24)
Southwest	139 (4)	112 (93 to 131)	144 (7)	116 (97 to 136)	4 (−21 to 28)
Interior	288 (9)	82 (72 to 91)	151 (7)	46 (38 to 53)	−44 (−59 to −29)
Northern	47 (1)	54 (38 to 70)	50 (2)	60 (43 to 77)	12 (−32 to 55)
Unknown	30 (1)	NC	41 (2)	NC	NC
Race**					
AI/AN	1,028 (32)	248 (233 to 264)	828 (38)	198 (184 to 212)	−20 (−29 to −12)
Asian	42 (1)	25 (17 to 32)	27 (1)	14 (8 to 19)	−45 (−83 to −8)
Black or African American	111 (3)	112 (90 to 135)	94 (4)	92 (72 to 111)	−18 (−45 to 8)
NH/PI	14 (<1)	15 (4 to 25)	37 (2)	29 (4 to 54)	97 (−86 to 280)
White	1,480 (46)	90 (85 to 94)	1,041 (48)	66 (62 to 71)	−26 (−33 to −19)
Other	241 (8)	NC	24 (1)	NC	NC
Unknown	322 (10)	NC	176 (8)	NC	NC
**Ethnicity^††^**					
Hispanic or Latino	94 (3)	63 (49 to 76)	108 (5)	70 (56 to 84)	12 (−20 to 43)
Not Hispanic or Latino	2,382 (74)	114 (110 to 119)	1,724 (80)	86 (82 to 90)	−25 (−30 to −19)
Unknown	722 (23)	NC	322 (15)	NC	NC

**Abbreviations:** AI/AN = American Indian or Alaska Native; NC= not calculated; NH/PI = Native Hawaiian or Pacific Islander.

* Annual rates were calculated using population estimates from the Alaska Department of Labor and Workforce. Direct age-standardization was performed using U.S. Census Bureau 2020 data as a reference population. Age-specific rates were not age-standardized.

^†^ Relative percentage change and 95% CI in age-standardized rates from 2016–2019 to 2020–2023 were calculated using a normal approximation for age-specific rates and a gamma approximation of the Poisson distribution for age-standardized rates.

^§^ All data are current as of May 21, 2024, and are subject to change, pending further updates.

^¶^ Urban was defined as residence in the Municipality of Anchorage, the Fairbanks North Star Borough, or the City and Borough of Juneau. Rural was defined as living in the Matanuska-Susitna or Kenai Peninsula Boroughs, which are on the road system. All other areas were considered remote.

** Race categories are not mutually exclusive: 112 (2%) persons selected two or more racial groups and are counted more than once in the analysis by racial group.

^††^ Race and ethnicity were reported separately and are not mutually exclusive (i.e., persons reporting Hispanic or Latino ethnicity could have identified as any race).

**FIGURE:**
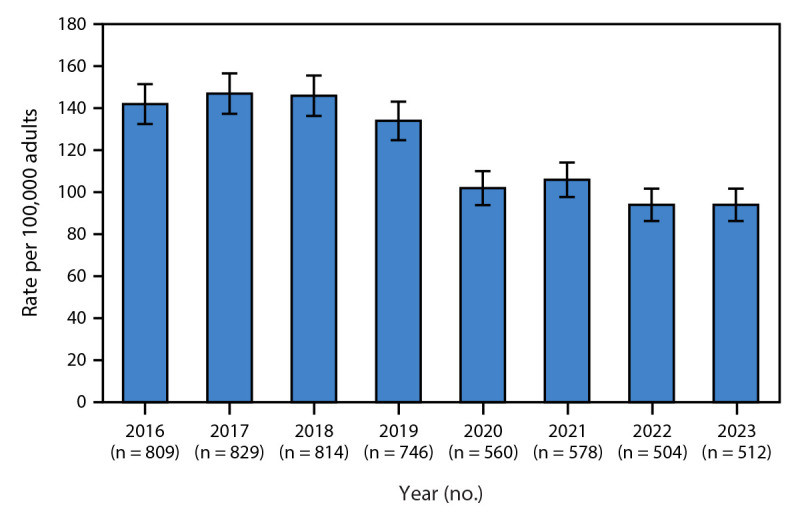
Annual age-standardized rate* and 95% CIs of newly reported chronic hepatitis C cases per 100,000 adults aged ≥18 years — Alaska, 2016–2023 * Annual rates were calculated using population estimates from the Alaska Department of Labor and Workforce Development. Direct age-standardization was performed using U.S. Census Bureau 2020 data as a reference population.

## Discussion

During 2016–2023, the average annual rate of newly reported chronic hepatitis C in Alaska was 121 cases per 100,000 adults. The overall rate decreased by 30% from 2016–2019 to 2020–2023, similar to national trends.^¶¶^ Similar to previous reports, groups with higher newly reported chronic hepatitis C rates included males, adults aged 18–39 years, American Indian or Alaska Native persons, and residents of rural areas.*** Most groups experienced decreases in chronic hepatitis C rates from 2016–2019 to 2020–2023, and no group experienced an increase.

Rates of newly reported chronic hepatitis C among Alaska adults aged <40 years during the surveillance period were approximately double the national rates reported during 2018 (*3*) and during 2019–2022.^†††^ Reported rates in Alaska by age group, region, and race might reflect differences in testing related to health care access or use. They also might reflect differences in exposure risk related to behavioral, environmental, or social factors (*6*).

Reasons for the decrease in newly reported chronic hepatitis C cases are likely multifactorial, including expanded hepatitis C screening and treatment and changes in health care–seeking, testing practices, or other behaviors, e.g., related to injection drug use or the COVID-19 pandemic. In 2020, national recommendations were expanded to include hepatitis C testing for all adults (*4*). However, the COVID-19 pandemic resulted in a temporary decrease in hepatitis C testing, treatment, and reporting (*7*). During 2021–2023, increases in opiate-related deaths occurred relative to previous years,^§§§^ and shifts in opioid use from injection to smoking were noted^¶¶¶^ (*8*); both of these factors might have contributed to reduced HCV transmission. Through the efforts of multiple partners in Alaska, hepatitis C screening and treatment has been expanded, including provision of direct-acting antiviral treatment through Medicaid.**** The impact of these efforts was corroborated in a recent analysis of data from a large commercial laboratory that estimated that 34% of persons with chronic hepatitis C in Alaska achieved viral clearance or cure during 2013–2022, similar to the national rate (the national target is ≥58% by 2025) (*9*).

In Alaska and nationally, challenges remain in ensuring that persons with reactive (positive) HCV antibody test results receive confirmatory testing for HCV RNA (current infection) and subsequent linkage to treatment, especially in remote areas. Efforts are needed to operationalize CDC’s updated 2023 guidance to use a single visit to obtain blood specimens for both steps of the HCV testing sequence (*10*). The point-of-care HCV RNA test, which was approved in June 2024, could help complement existing test-and-treat strategies, such as for populations in remote areas without road access and for those who use injection drugs.^††††^

State program goals are to expand hepatitis C screening and linkage to care among disproportionately affected groups, monitor progress through the HCV clearance cascade (from testing to treatment and cure), and improve perinatal hepatitis C surveillance and prevention. Reporting of negative HCV RNA results to the state was instituted in September 2023,^§§§§^ which should improve case classification and allow monitoring of testing access and viral clearance and cure. The updated reportable conditions statute also includes provider reporting of pregnancy status among women with hepatitis C, which will improve monitoring to guide perinatal hepatitis C prevention.^¶¶¶¶^ The Alaska Hepatitis Advisory Working Group of state partners meets quarterly and is drafting a state plan for viral hepatitis elimination, in alignment with national elimination efforts (J Allison, MPH, Alaska Department of Health, personal communication, July 2024).

### Limitations

The findings in this report are subject to at least four limitations. First, the reported rates are those of cases newly reported to Alaska by providers and laboratories; the data do not distinguish incident (new) from prevalent infection and would include persons who received positive test results before 2016 but still need treatment, and persons with hepatitis C who have not received testing. Second, the number and rate of chronic hepatitis C cases might be underestimated because only confirmed cases are included. Third, some acute hepatitis C cases might have been misclassified as chronic cases because of missing clinical information and limited reporting of negative HCV RNA test results. Finally, the small size of some demographic groups and changes in the completeness of race and ethnicity data over time likely limited the ability to assess changes in some rates.

### Implications for Public Health Practice

Hepatitis C surveillance can help monitor health outcomes and identify groups needing tailored testing and treatment interventions aimed toward hepatitis C elimination. Further research is needed to better understand factors that contribute to HCV transmission (e.g., differences in access to testing and treatment); additional efforts are needed to improve access to hepatitis C prevention, testing, and treatment services.

## Footnotes

*
https://www.hhs.gov/sites/default/files/Viral-Hepatitis-National-Strategic-Plan-2021-2025.pdf

†
https://www.cdc.gov/nbs/php/index.html

§ This definition is based on the 2020 confirmed chronic hepatitis C case definition (https://ndc.services.cdc.gov/case-definitions/hepatitis-c-chronic-2020/), which does not substantively differ from the 2016 definition (https://ndc.services.cdc.gov/case-definitions/hepatitis-c-chronic-2016/) in ways that might affect surveillance reporting over time.

¶ Urban was defined as residence in the Municipality of Anchorage, the Fairbanks North Star Borough, or the City and Borough of Juneau. Rural was defined as living in the Matanuska-Susitna or Kenai Peninsula Boroughs, which are on the road system. All other areas were considered remote.

**
https://live.laborstats.alaska.gov/data-pages/alaska-population-estimates

††
https://www.census.gov/data/datasets/time-series/demo/popest/2020s-national-detail.html

§§ 45 C.F.R. part 46.102(l)(2), 21 C.F.R. part 56; 42 U.S.C. Sect.241(d); 5 U.S.C. Sect.552a; 44 U.S.C. Sect. 3501 et seq.

¶¶ National chronic hepatitis C rates decreased from an average of 54 per 100,000 persons of all ages during 2009–2018 to 40 per 100,000 during 2020–2022. https://www.cdc.gov/hepatitis/php/statistics-surveillance/?CDC_AAref_Val

*** https://epi.alaska.gov/bulletins/docs/b2016_19.pdf; https://epi.alaska.gov/bulletins/docs/b2019_15.pdf; https://epi.alaska.gov/bulletins/docs/b2013_13.pdf

††† https://archive.cdc.gov/www_cdc_gov/hepatitis/statistics/2019surveillance/Table3.6.htm; https://www.cdc.gov/hepatitis/statistics/2020surveillance/hepatitis-c/table-3.6.htm;

§§§ https://www.cdc.gov/nchs/nvss/vsrr/drug-overdose-data.htm; https://health.alaska.gov/dph/VitalStats/Documents/PDFs/DrugOverdoseMortalityUpdate_2022.pdf

¶¶¶
https://www.cdc.gov/overdose-prevention/data-research/facts-stats/sudors-dashboard-fatal-overdose-data.html

**** https://vimeo.com/1061082524; https://nastad.org/sites/default/files/2024-05/Robert%20Lawrence%20-%20Hepatitis%20C%20Elimination%20Alaska%20DOC.pdf

††††
https://stacks.cdc.gov/view/cdc/164804

§§§§
https://health.alaska.gov/dph/Epi/Documents/pubs/conditions/ConditionsReportable_LABS.pdf

¶¶¶¶
https://health.alaska.gov/dph/Epi/Documents/pubs/conditions/ConditionsReportable_HCP.pdf

## References

[1] Lewis KC, Barker LK, Jiles RB, Gupta N. Estimated prevalence and awareness of hepatitis C virus infection among US adults: National Health and Nutrition Examination Survey, January 2017–March 2020. Clin Infect Dis 2023;77:1413–5. 10.1093/cid/ciad411 37417196 PMC11000503

[2] Bressler SS, Bruden D, Nolen LD, Mortality among Alaska Native adults with confirmed hepatitis C virus infection compared with the general population in Alaska, 1995–2016. Can J Gastroenterol Hepatol 2022;2022:2573545. 10.1155/2022/2573545 35178364 PMC8847038

[3] Ryerson AB, Schillie S, Barker LK, Kupronis BA, Wester C. Vital signs: newly reported acute and chronic hepatitis C cases—United States, 2009–2018. MMWR Morb Mortal Wkly Rep 2020;69:399–404. 10.15585/mmwr.mm6914a2 32271725 PMC7147907

[4] Schillie S, Wester C, Osborne M, Wesolowski L, Ryerson AB. CDC recommendations for hepatitis C screening among adults—United States, 2020. MMWR Recomm Rep 2020;69(RR-2):1–17. 10.15585/mmwr.rr6902a1 32271723 PMC7147910

[5] Townshend-Bulson L, Roik E, Barbour Y, The Alaska Native/American Indian experience of hepatitis C treatment with sofosbuvir-based direct-acting antivirals. PLoS One 2021;16:e0260970. 10.1371/journal.pone.0260970 34855920 PMC8639063

[6] Craib KJ, Spittal PM, Patel SH, ; Cedar Project Partnership. Prevalence and incidence of hepatitis C virus infection among Aboriginal young people who use drugs: results from the Cedar Project. Open Med 2009;3:e220–7. 21688759 PMC3090112

[7] Kaufman HW, Bull-Otterson L, Meyer WA 3rd, Decreases in hepatitis C testing and treatment during the COVID-19 pandemic. Am J Prev Med 2021;61:369–76. 10.1016/j.amepre.2021.03.011 34088556 PMC8107198

[8] Tanz LJ, Gladden RM, Dinwiddie AT, Routes of drug use among drug overdose deaths—United States, 2020–2022. MMWR Morb Mortal Wkly Rep 2024;73:124–30. 10.15585/mmwr.mm7306a2 38358969 PMC10899081

[9] Tsang CA, Tonzel J, Symum H, State-specific hepatitis C virus clearance cascades—United States, 2013–2022. MMWR Morb Mortal Wkly Rep 2024;73:495–500. 10.15585/mmwr.mm7321a4 38814852 PMC11152369

[10] Cartwright EJ, Patel P, Kamili S, Wester C. Updated operational guidance for implementing CDC’s recommendations on testing for hepatitis C virus infection. MMWR Morb Mortal Wkly Rep 2023;72:766–8. 10.15585/mmwr.mm7228a2 37440452 PMC10360608

